# The Regulatory Effect of Histone Deacetylase (HDAC) 1 and 2 on iNOS, IL‐6, TNF‐α and IL‐10 Expression in Canine Macrophages Infected With *Leishmania infantum*

**DOI:** 10.1111/pim.70078

**Published:** 2026-04-06

**Authors:** Gabriela Lovizutto Venturin, Lucas Takeshi Siqueira Ito, Gisele Mitsue Umino, Gabriela Torres Rebech, Flávia Lombardi Lopes, Valéria Marçal Felix de Lima

**Affiliations:** ^1^ Department of Clinical Medicine, Surgery and Animal Reproduction São Paulo State University (UNESP), School of Veterinary Medicine Araçatuba São Paulo Brazil; ^2^ Department of Production and Animal Health São Paulo State University (UNESP), School of Veterinary Medicine Araçatuba São Paulo Brazil

## Abstract

Zoonotic visceral leishmaniasis is caused by *Leishmania (Leishmania) infantum*. Dogs are considered the most critical urban reservoirs of *L. (L.) infantum* due to their high infection rate and direct transmission to humans. The parasite has developed mechanisms to evade the host's defence system by inhibiting macrophage activation, thereby allowing it to replicate and survive. Pathogens such as viruses and bacteria can modify the host's epigenome, thereby facilitating their survival. Cellular reprogramming leads to epigenetic alterations that modulate chromatin, modify histones and DNA methylation, disrupt normal progression and compromise the continuity of cell differentiation. Histone deacetylases (HDACs) remove lysine acetyl groups from histones, resulting in chromatin alteration and gene silencing. Histone acetyltransferases regulate their function. In this study, we analysed the involvement of HDAC‐1 in the defen mechanisms of DH82 macrophages infected with *L. infantum*. We observed that *L. infantum* infection increases HDAC1 levels and expression. Silencing HDAC1 with siRNA and the pharmacological inhibitor NaB decreased parasite load and increased iNOS expression, associated with increased histone acetylation at the iNOS promoter. The pharmacological inhibitor NaB also decreased IL‐6, IL‐10 and TNF‐α levels in the culture supernatant of DH82 macrophages infected with *L. infantum*. Together, these findings indicate that *L. infantum*‐induced HDAC1 upregulation modulates the expression of key innate immune response genes, contributing to the establishment of infection in canine macrophages.

## 1 Introduction

Zoonotic visceral leishmaniasis is caused by *Leishmania (Leishmania) infantum*. It is endemic in the Americas, Europe and Asia, with new areas of dissemination being identified regularly. In the Americas, transmission between vertebrate hosts occurs through the bite of the hematophagous female phlebotomine *Lutzomyia longipalpis*. Dogs ( *Canis familiaris* ) are the most important urban reservoirs of *L. infantum* due to their high infection and transmission rates to humans [1]. Dogs are highly susceptible to infections, and current treatments have shown limited efficacy; therefore, knowledge of the immune response is fundamental. This parasite species multiplies inside host macrophages in the liver, spleen and bone marrow, and about 90% of infected dogs can remain asymptomatic or subclinical for years [2].

Innate immunity is the first line of defence against the protozoan [3]. *Leishmania* antigens are recognised by receptors on dendritic cells, natural killer cells, monocytes/macrophages and neutrophils [4]. Among these receptors are Toll‐like receptors, which use adapter proteins such as MyD88 to activate transcription factors NF‐kB and IRF, thereby increasing the expression of nitric oxide synthase (iNOS) in the infected macrophage, an enzyme that produces nitric oxide (NO) [5, 6, 7]. This canonical NF‐kB pathway is also responsible for transcriptional induction of a large number of inflammatory genes, including those encoding TNF‐α, IL‐1β, IL‐6, IL‐12p40, chemokines and additional inflammatory mediators in macrophages [8].

NO is an important effector molecule directly involved in microbicidal and cytotoxic activity in humans [9]. In splenic leukocytes from dogs, NO is produced in response to infection by *L. infantum* [10]. Nitric oxide production was also observed in the supernatant of canine macrophages infected in vitro with *L. infantum* promastigotes [11] and may be involved in protection against natural infection by *Leishmania* spp. [12].

The parasite has developed mechanisms to evade the host's defence system by inhibiting macrophage activation, thereby allowing it to replicate and survive. Studies show that various pathogens, such as viruses and bacteria, affect the host's epigenetic mechanisms, thus facilitating their survival [13, 14]. Epigenetic modifications that have been identified as being induced by Leishmania infection include chromatin remodelling [15], DNA methylation [16] and modifying histones [17].

Enzymes that alter histones are essential for regulating cell chromatin structure and gene expression in eukaryotic organisms. Acetylation of nucleosome histone proteins H2A, H2B, H3 and H4, mediated by histone acetyltransferases and HDACs, is one of the post‐translational modifications of histones that has been studied the most. Increased acetylation by histone acetyltransferase proteins is associated with active transcription [18]. On the other hand, HDACs are enzymes that remove acetyl groups from histone lysines, thereby remodelling chromatin and promoting gene silencing [19].

Histone deacetylase ‐ 1 regulates the transcription of proteins involved in immunity. The interaction of HDAC‐1 with p50 homodimers bound at the kB site of promoters and enhancers constitutes a key mechanism that constrains NF‐kB‐dependent transcription of at least a subset of inflammatory genes [20]. Because of the crucial role of HDACs in immunoregulation, inhibitors are under investigation as potential treatments for various diseases, including cancer, HIV/AIDS and other parasitic infections [21]. The potential of HDAC inhibitors as antiparasitic agents was first observed more than 10 years ago, when it was discovered that the cyclic tetrapeptide apicidin has broad‐spectrum antiparasitic activity. Since then, many studies have focused on the antiparasitic function of HDAC inhibitors of various structural classes, highlighting the potential of these drugs for antiparasitic intervention [18, 22].

Some studies have shown that HDAC1 plays a role in immunity in leishmaniasis. Hypoacetylation of H3 was associated with negative regulation of inflammatory cytokines (IL‐1 and TNF‐α), phenotypically altering murine macrophages infected with *L. amazonensis* towards an anti‐inflammatory profile, beneficial for parasite survival [23]. Changes in HDAC1 activity can also affect macrophage polarisation by shifting them from a pro‐inflammatory to an anti‐inflammatory phenotype [24, 25], but the role of HDAC1 in macrophage polarisation infected with *L. infantum* has not yet been investigated.

In human macrophages infected with *L. donovani* , silencing HDAC1 decreased intracellular parasite survival [17]. In vitro, infection of human macrophages with *L. amazonensis* increased HDAC1 expression and contributed to parasite survival by negatively regulating NO production and favouring disease progression [26]. Dogs with visceral leishmaniasis may show progressive disease, which could indicate a failure in innate immunity; the evaluation of immune regulatory mechanisms associated with HDACs has not been investigated.

In the present study, we investigated whether infection of canine DH82 macrophages with *L. infantum* affects HDAC1 levels and whether HDAC1 modulates iNOS, IL‐6, TNF‐α and IL‐10 expression, as well as parasite survival after infection.

## 2 Methods

### 2.1 Macrophage Infection

The Canine DH82 macrophages, kindly supplied by Prof. Dr. Felipe da Silva Krawczak (Escola de Veterinária e Zootecnia da Universidade Federal de Goiás (UFG), GO, Brazil). Cell line infection was performed in 24‐well culture plates at 2.0 × 10^5^ macrophages/well in a total volume of 300 μL, with the assay performed in triplicate. The cells were infected with promastigote forms of *L. infantum (MHOM/BR/00/MERO2)* at a macrophage/parasite ratio of 1:15 in RPMI‐1640 medium (Sigma‐Aldrich, MO, USA), supplemented with 10% heat‐inactivated fetal bovine serum (Thermo Fisher Scientific, MA, USA), 0.03% l‐glutamine (Sigma‐Aldrich, MO, USA) and 100 μL/mL penicillin (Sigma‐Aldrich, MO, USA) and 100 mg/mL streptomycin (Sigma‐Aldrich, MO, USA) (RPMI 1640‐complete). The cultures were maintained at 37°C, 5% CO_2_. After 6 h of incubation, the cells were recovered with cell scrapers. The HDAC1 transcript level was higher at this time than at 12 and 24 h post‐infection (data not shown). Microscopic slides were prepared following in vitro infection with *Leishmania*. DH82 cells were stained with a commercial hematologic stain (Rapid Panoptic 3X500ML, Laborclin), and the infection was examined at oil‐immersion magnification on an Olympus BX61 microscope equipped with a DP71 camera. *Leishmania infantum* were observed inside DH82 cells (Figure S1).

### 2.2 HDAC1 and iNOS Expression by Flow Cytometry

The infected macrophages were fixed with 500 μL of fixation buffer (Invitrogen, MA, USA) for 10 min at room temperature. The cells were centrifuged at 1800 rpm for 5 min and washed twice with 500 μL permeabilisation buffer (Invitrogen, MA, USA). The cells were then resuspended in 50 μL of permeabilisation buffer (Invitrogen, MA, USA) and labelled with anti‐human HDAC1‐FITC‐conjugated antibody (Santa Cruz Biotechnology, TX, USA). The human HDAC1 protein (NP_004955.2) blast is 93.75% similar to the canine HDAC1 protein (XP_038387161.1). For iNOS labelling, the same protocol was followed; however, after resuspending the cells in 50 μL of permeabilisation buffer (Invitrogen, MA, USA), they were labelled with PE‐conjugated human anti‐iNOS antibodies (BIORBYT, Cambridge, UK) and their respective isotype controls (BIORBYT, Cambridge, UK). The human iNOS protein (NP_000616.3) blast is similar to the canine iNOS protein (NP_001300777.1) of 85.81%.

Whole cells were distinguished from fragments by gating based on the forward and side scatter signals. Fluorescence intensity was measured using BD Accuri C6 software (BD Biosciences, CA, USA). Data acquisition and analysis were performed with 10,000 closed events in BD Accuri C6 software, Version 1.0.264.21 (BD Biosciences, CA, USA).

### 2.3 Measurement of NO_2_ Levels

Nitrite levels in the supernatants of cultures were determined using a standard Griess reagent. Briefly, 100 μL of supernatant from macrophage cultures was mixed with an equal volume of Griess reagent containing 1% sulphanilamide (Sigma‐Aldrich) diluted in 5% H_3_PO_4_ and 0.1% N‐(1‐naphthyl)‐ethylenediamine (Sigma‐Aldrich). After a 10‐min incubation at room temperature, the plates were read on a spectrophotometer (Packard Bio Science Company) with a 540 nm filter. The NO_2_ concentration was determined by comparison of the obtained values with those obtained using a standard curve of 0.78–200 μmol/L nitrite (NO_2_).

### 2.4 Endogenous HDAC1 and HDAC2 Suppression

To suppress endogenous HDAC1 and HDAC2 expression, the cells were treated for 6 h with NaB (Sigma‐Aldrich, MO, USA) (10 mM) and (20 mM) after *L. infantum* infection. The inhibitor was used according to the manufacturer's instructions. Low butyrate concentrations could inhibit class 1 and 2 HDACs [27].

### 2.5 Transfection With siRNA

For HDAC1 inhibition, macrophages were incubated with interference canine RNAs: Sc‐siRNA (negative control), HDAC1‐siRNA (s150968) and HDAC1‐siRNA2 (s119558) (Thermo Fisher Scientific, MA, USA) for 24 h to allow silencing, according to the manufacturer's instructions. The cells were then washed and incubated with *L. infantum* 1:15. Cells were harvested 6 h after infection, and RNA was extracted, followed by cDNA synthesis. The expression of HDAC1 and iNOS mRNAs was analysed by qRT‐PCR.

### 2.6 Chromatin Immunoprecipitation (ChIP)

Chromatin immunoprecipitation was conducted using the commercial ChromaFlash One‐Step Magnetic ChiP kit (Epigentek, NY, USA), using the Histone H3 (1B1B2) at a concentration of 1:50 (Cell Signalling, MA, USA) and the Isotype control (Mouse IgG1) at a concentration of 1:100 (Cell Signalling, MA, USA). Two replicates were performed, and the protocol was carried out according to the manufacturer's recommendations.

### 2.7 HDAC1 Activity Test

Following the manufacturer's recommendations, HDAC1 protein activity was determined using the Epigenase HDAC Activity/Inhibition Direct Assay Kit (Colorimetric) (Epigentek, NY, USA). The sensitivity was 0.5 ng/μL.

### 2.8 Cell Fractionation

Nuclear extracts from DH82 cells infected and not infected with *L. infantum* (4 × 10^8^ cells) were prepared using a Rockland assay protocol. The harvested cells were washed gently with PBS and centrifuged at 1000 rpm for 5 min. The pellets were resuspended in five pellet volumes of cytoplasmic extract (CE) buffer containing 10 mM HEPES–KOH, pH 7.6, 60 mM KCl, 1 mM EDTA, 0.075% NP40, 1 mM DTT and PMSF 1 mM to isolate the cytoplasmic extract. After incubation on ice for 3 min, the samples were centrifuged at 1500 rpm for 4 min. The CE was collected and stored. The nuclear sediment was washed with detergent‐free CE buffer and centrifuged at 1500 rpm for 4 min. A sediment volume nuclear extract (NE) buffer containing 20 mM Tris–Cl, pH 8.0, 1.5 mM MgCl_2_, 420 mM NaCl, EDTA 0.2 mM and 25% glycerol was used to resuspend the nuclear sediment. The salt concentration was adjusted to 400 mM using 5 M NaCl. An additional pellet volume of NE buffer was added. The nuclear pellets in NE buffer were incubated on ice for 10 min with periodic shaking. The samples were then centrifuged at maximum speed for 10 min to sediment any irrelevant cell debris, and the extracts were stored at −80°C with 20% glycerol.

### 2.9 RNA Extraction and Quantitative RT‐PCR (qRT‐PCR)

According to the manufacturer's recommendations, total RNA from infected macrophages was isolated using the commercial RNeasy Mini Kit (Quiagen, CA, USA). The RNA was then eluted in nuclease‐free water, and the concentration of the extracted RNA (ng/μL) and the degree of purity (A260 nm/A280 nm coefficient) were assessed using a spectrophotometer (NanoDrop Technologies ND‐1000 UV–Vis, USA). The samples were frozen at −80°C for the subsequent reverse transcription reaction. The cDNA was produced using the commercial QuantiTect Reverse Transcription kit (Qiagen, CA, USA) with 100 ng of RNA and oligo(dT) primers in a final volume of 20 μL. The cDNA was then frozen at −20°C until analysis. The qRT‐PCR reactions were standardised with 50 ng of cDNA, 4 μL of 5× HOT FIREPol Evagreen qPCR Supermix (Solis BioDyne, Tartu, Estonia), 10 pmol of each oligonucleotide primer and 13 μL of ultrapure water, for a final reaction volume of 20 μL. The amplification conditions were an initial incubation of 12 min at 95°C, followed by 40 cycles at 95°C for 15 s, 65°C for 25 s and 72°C for 25 s. After amplification, a dissociation curve of the amplified fragment was determined under the following conditions: 95°C for 15 s, 60°C for 15 s, followed by 20 min until reaching 95°C for 15 s. Nuclease‐free water (Sigma‐Aldrich, MO, USA) was used as a negative control for the reaction, and the samples were evaluated in triplicate. The reaction efficiency values, coefficients of determination (*r* ^2^) and angular coefficients (slopes) were obtained from the amplification of seven serial dilutions of a set of cDNA. Gene expression was quantified using the 2‐ΔΔCt method according to [28]. The values were normalised using the geometric mean of the gene expression of the reference genes Beta‐actin (5′CCAGCAAGGATGAAGATCAAG3′ and 5′TCTGCTGGAAGGTGGACAG3′) and HPRT‐1 (5′CACTGGGAAAACAATGCAGA3′ and 5′ACAAAGTCAGGTTTATAGCCAACA3′) [29] and represented by relative gene expression.

### 2.10 Quantification of the Cytokine IL‐6, TNF‐α and IL‐10 by Capture ELISA

Cytokines IL‐6, TNF‐α and IL‐10 were quantified in culture supernatant from *L. infantum* infected macrophages after HDAC1 and HDAC2 suppression with NaB (10 mM) and (20 mM) (Sigma‐Aldrich, MO, USA) by using the DuoSet ELISA Development Systems canine kits (R&D Systems, Minneapolis, MN, USA), following the manufacturer's instructions.

### 2.11 Quantification of Parasites in Cell Culture by Light Microscopy

For light microscopy evaluation after 6 h of culture, the cells were cytocentrifuged (Microprocessed Cytological Centrifuge, 2000 D, REVAN, Chientec) at 1000 rpm for 5 min at room temperature. The resulting material was used to prepare slides, which were stained with a commercial hematologic stain (Rapid Panoptic 3X500ML, Laborclin) for parasite counting in infected macrophages under an optical microscope (Eclipse E800, Nikon, Tokyo, JP). The infection index was determined by analysing 100 infected macrophages and calculating the ratio of total amastigotes to infected macrophages.

### 2.12 Statistical Analysis

Statistical analysis was done using GraphPad Prism v6 software (GraphPad Software Inc., La Jolla, CA, USA). All statistical variables were tested for normality using the Shapiro–Wilk test. Where it showed a non‐parametric distribution. The Wilcoxon test and Friedman test, followed by Dunn's multiple‐comparisons test, were used to compare results across groups. Values were considered significant when *p* < 0.05.

## 3 Results

### 3.1 Expression and Activity of HDAC1 in Cells Infected With *L. infantum*

**FIGURE 1:**
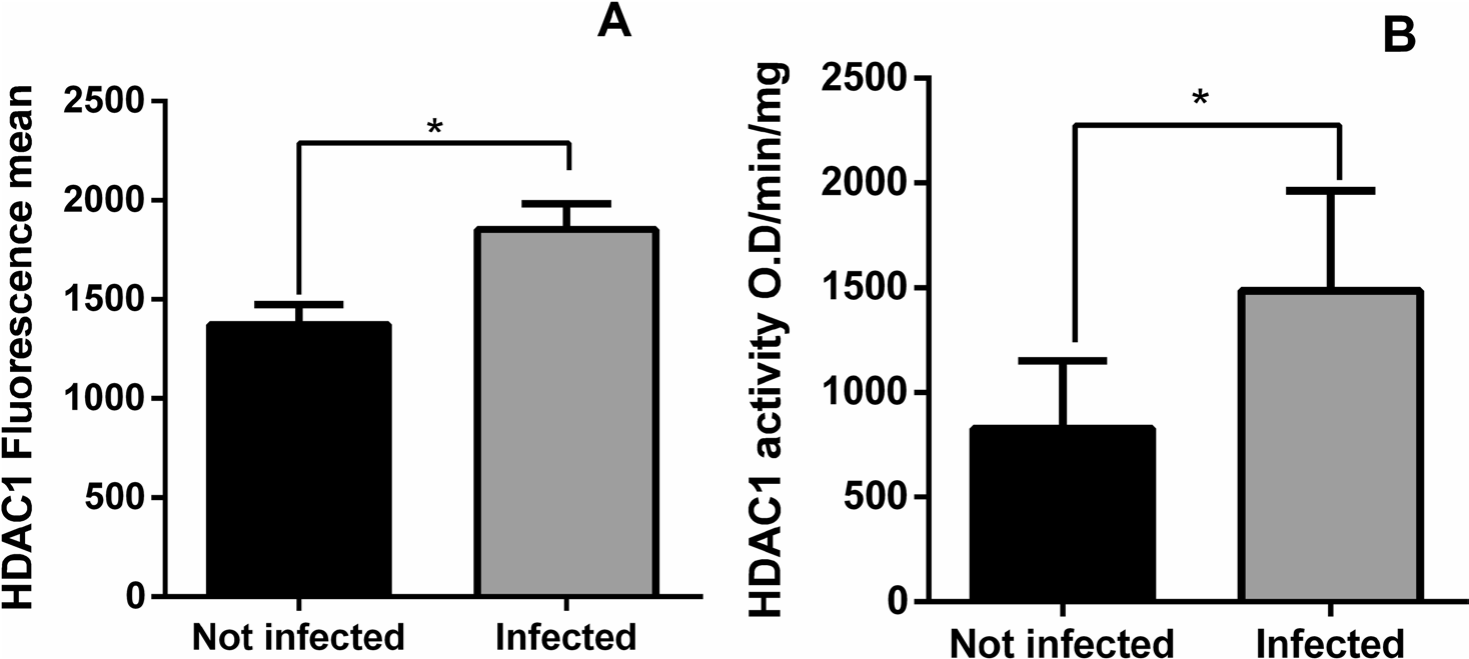
Expression and activity of HDAC1 in DH82 cells infected or not with *Leishmania infantum*. DH82 cells (2 × 10^5^ cells/well) were infected with *L. infantum* (1:15 MOI). Cells were harvested 6 h after infection. (A) HDAC1 expression was measured by flow cytometry in infected and uninfected cells. (B) After 6 h of infection, cell lysates from infected and uninfected cells were used to measure HDAC1 enzyme activity using a colorimetric kit. The colorimetric unit was calculated as described in the methods. The statistical test used was Wilcoxon (*p* < 0.05). The graphs are represented with the mean and standard error of the mean.

Increased expression of HDAC1 and its activity was observed in human macrophages infected with *L. donovani* [17]. Therefore, we evaluated the expression and activity of this enzyme in DH82 cells infected with *L. infantum*. DH82 cells were infected with *L. infantum* (1:15), and after 6 h, the cells were harvested. Increased expression (1373.5 ± 209.5 (not infected); 1853.5 ± 254.8 (infected); Wilcoxon test, *p = 0.0313) (Figure 1A) and activity (828.8 ± 711.5 (not infected); 1487.1 ± 1012.8 (infected), Wilcoxon test, *p = 0.0313) (Figure 1B) of HDAC1 were observed in cells infected with *L. infantum* compared to uninfected cells. A representative histogram of HDAC1 expression in DH82 cells not infected and infected with *L. infantum* is shown in (Figure S2).

### 3.2 Silencing and Pharmacological Inhibition of HDAC1 and HDAC2 Reduce Infection Index

The HDAC1‐siRNA1 and HDAC1‐siRNA2 silencers and the pharmacological HDAC1 and HDAC2 inhibitor NaB were used to assess whether HDAC1 expression regulates parasite load. DH82 cells were transfected with Sc‐siRNA, HDAC1‐siRNA1 and HDAC1‐siRNA2 24 h before infection with *L. infantum*. After 6 h of infection, the transfected cells were harvested and HDAC1 expression was analysed by flow cytometry. We observed that HDAC1 expression decreased in cells transfected with HDAC1‐siRNA1 (Figure S3A) and HDAC1‐siRNA2 (Figure S3B) compared to cells transfected with Sc‐siRNA. This result confirms the effect of specific silencing on HDAC expression in DH82 cells.

DH82 cells were treated with the pharmacological inhibitor NaB (20 mM), infected with *L. infantum for 6 h*, and HDAC‐1 expression was assessed. We observed a decrease in HDAC1 expression in the presence of the pharmacological inhibitor NaB compared to the medium (Figure S3C).

**FIGURE 2:**
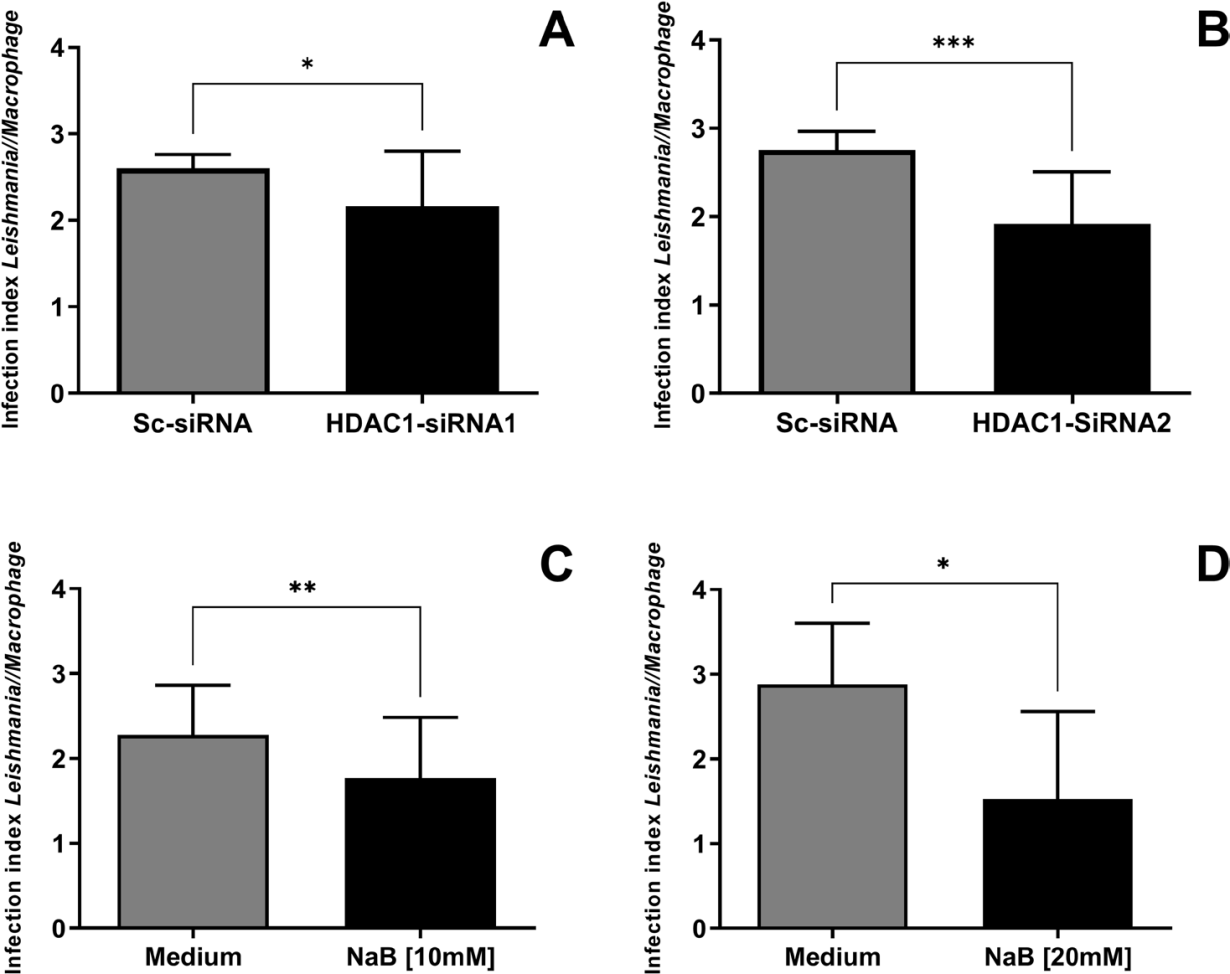
Regulation of HDAC1 action on infection index in DH82 cells infected with *Leishmania infantum*. Infection index (1:15) in DH82 cells transfected with (A) Sc‐siRNA and HDAC1‐si‐RNA1 and with (B) Sc‐siRNA and HDAC1‐si‐RNA2. Infection Index (1:15 MOI) in DH82 cells infected with *L. infantum* and treated with the pharmacological inhibitor NaB (C) (10 mM) and (D) (20 mM). The statistical test used was the Wilcoxon test (*p < 0.05, **p < 0.01, ***p < 0.001). The graphs are represented with the mean and standard error of the mean.

Next, we evaluated the parasite load in DH82 cells infected with *L. infantum*, transfected with siRNAs, or treated with the pharmacological inhibitor NaB. The infection index was analysed by counting the total number of amastigotes within macrophages and dividing by 100 of infected DH82 cells. We observed a decrease in infection index in cells transfected with HDAC1‐siRNA1 (2.6 ± 0.3 (Sc‐siRNA); 2.1 ± 0.4 (HDAC1‐siRNA1), Wilcoxon test, *p = 0.0371) (Figure 2A) and in those transfected with HDAC1‐siRNA2 (2.7 ± 0.4 (Sc‐SiRNA); 1.9 ± 0.4 (HDAC1‐siRNA2), paired *t*‐test, ***p = 0.0001) (Figure 2B) compared to cells transfected with Sc‐siRNA. The decrease of infection index was also observed in DH82 cells infected with *L. infantum* and treated with the pharmacological inhibitor NaB (10 mM) (2.2 ± 0.4 (Medium); 1.7 ± 0.4 (NaB [10 mM]), Wilcoxon test, ***p = 0.0011) (Figure 2C) and (20 mM) (2.8 ± 0.5 (Medium); 1.5 ± 0.7 (NaB [20 mM]), Wilcoxon test, *p = 0.0313) (Figure 2D). Representative slides of infection index showing *Leishmania infantum* inside DH82 cells are shown (Figure S4A,B).

### 3.3 Silencing and Pharmacological Inhibition of HDAC1 and HDAC2 Increase iNOS Expression and NO_2_ Levels

**FIGURE 3:**
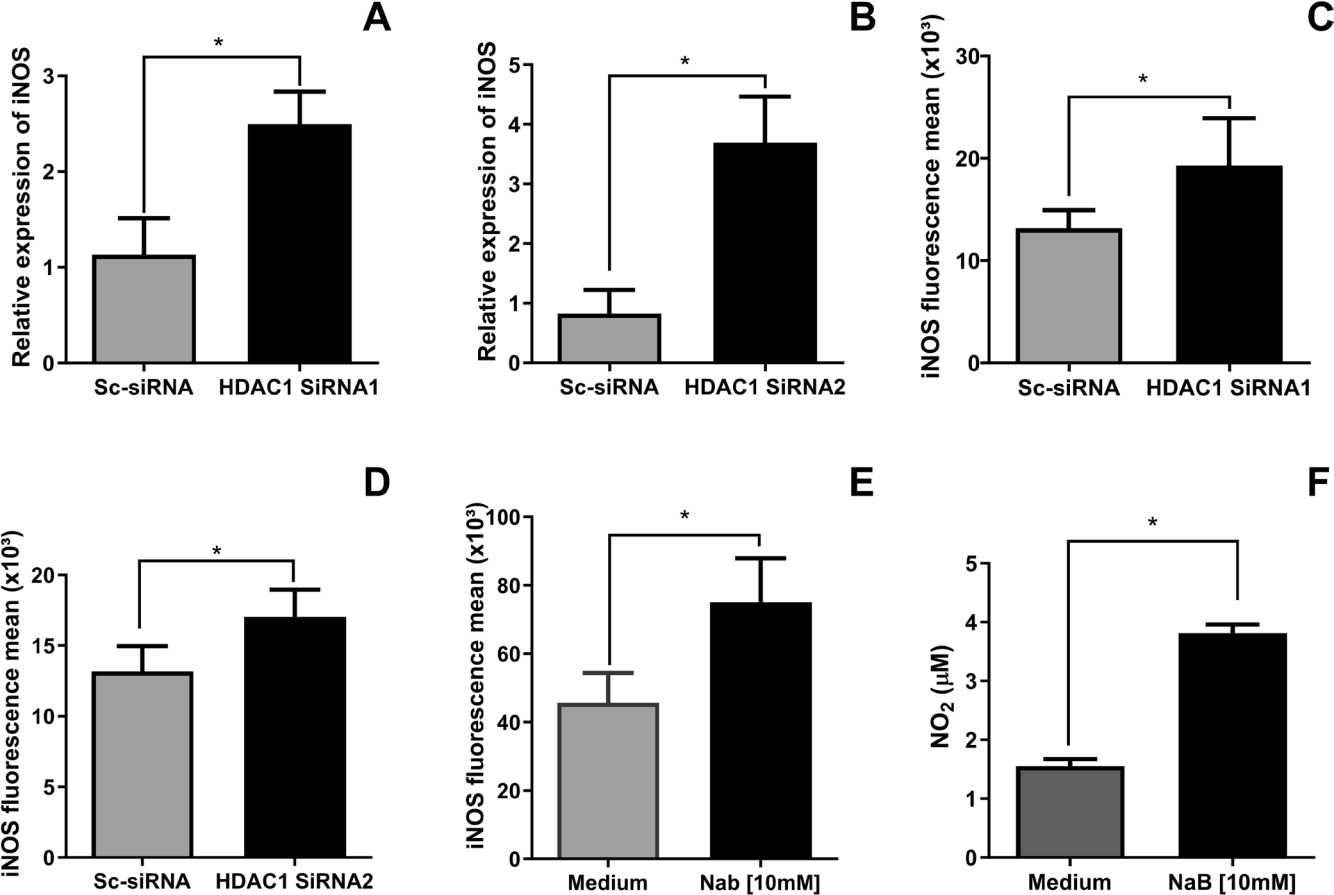
Silencing of HDAC1 in the iNOS defense gene in cells infected with *Leishmania infantum* and action of sodium butyrate on iNOS expression. DH82 cells were transfected with siRNA after 24 h and infected with *L. infantum*. The cells were harvested, RNA was extracted and qRT‐PCR was performed. The cells were treated with the pharmacological HDAC1 inhibitor sodium butyrate NaB (10 mM) and infected. After infection, the cells were harvested and read by flow cytometry. Relative expression of iNOS in DH82 cells infected with *L. infantum* and transfected with (A) Sc‐siRNA and HDAC1‐si‐RNA1 and with (B) Sc‐siRNA and HDAC1‐si‐RNA2. The levels of iNOS were assessed in DH82 cells transfected with siRNA for 24 h, followed by infection with *L. infantum*. The cells were harvested, and iNOS levels were measured by flow cytometry. Average iNOS fluorescence in DH82 cells transfected with (C) Sc‐siRNA and HDAC1‐si‐RNA1, (D) Sc‐siRNA and HDAC1‐si‐RNA2 and with (E) pharmacological inhibitor NaB [10 mM]. (F) NO_2_ levels in DH82 cells infected with *L. infantum* and inhibited with NaB (10 mM). The statistical test used was Wilcoxon (*p* < 0.05). The graphs are represented with the mean and standard error of the mean.

In canine leishmaniasis, high iNOS expression in macrophages is associated with parasite elimination, resulting in a lower intracellular parasite load [30]. Therefore, we determined whether HDAC1 is involved in iNOS expression. DH82 cells were transfected with Sc‐siRNA, HDAC1‐siRNA and HDAC1‐siRNA2 24 h before infection with *L. infantum*. Cells were harvested 6 h after infection, followed by RNA isolation, cDNA synthesis, qRT‐PCR for iNOS and iNOS protein analysis by flow cytometry. HDAC1 silencing with HDAC1‐siRNA1 (1.1 ± 0.6 (Sc‐siRNA); 2.4 ± 0.6 (HDAC1‐siRNA1), Wilcoxon test, *p = 0.0313) and HDAC1‐siRNA2 (0.8 ± 0.5 (Sc‐siRNA); 3.7 ± 0.9 (HDAC1‐siRNA2), paired *t*‐test, *p = 0.0297) increased iNOS mRNA levels (Figure 3A,B) and iNOS expression (13.1 ± 3.5 (Sc‐siRNA); 19.3 ± 8.3 (HDAC1‐siRNA1), Wilcoxon test, *p = 0.0313); (13.1 ± 3.5 (Sc‐siRNA); 17.0 ± 4.0 (HDAC1‐siRNA2), paired *t*‐test, *p = 0.0479) (Figure 3C,D).

Because silencing HDAC1 increases iNOS levels, we assess whether the pharmacological inhibitor increases iNOS and NO_2_ following sodium butyrate treatment, a known HDAC1 and HDAC2 inhibitor. DH82 cells were infected with *L. infantum for 6 h* and treated with the pharmacological NaB. The expression of iNOS was assessed by flow cytometry. NO_2_ levels in the supernatant were measured using the Griess reagent and read at 540 nm using a spectrophotometer. We observed increased iNOS (45.6 ± 15.4 (Medium); 75.1 ± 9.3 (NaB [10 mM]), paired *t*‐test, **p = 0.0036) (Figure 3E) and NO_2_ (1.5 ± 0.3 (Medium); 3.8 ± 0.4 (NaB [10 mM]), paired *t*‐test, ****p < 0.0001) (Figure 3F) levels in NaB‐treated cells. A representative histogram of iNOS production in DH82 cells infected with *L. infantum*, transfected with HDAC1‐siRNA (Figure S5A), HDAC1‐siRNA2 (Figure S5B) and treated with NaB (Figure S5C).

### 3.4 *Leishmania infantum* Affects Histone Remodelling in the iNOS Gene Promoter by Remodelling Chromatin Through Epigenetic Regulation

Post‐translational histone changes, such as acetylation and deacetylation, alter the chromatin structure, making it difficult for transcription factors to access essential genes during an immune response [27]. Decreased acetylation in H3 (Ac‐H3) residues associated with promoter genes of the NF‐kB pathway, responsible for iNOS production, may be associated with a reduced response against the parasite [23].

**FIGURE 4:**
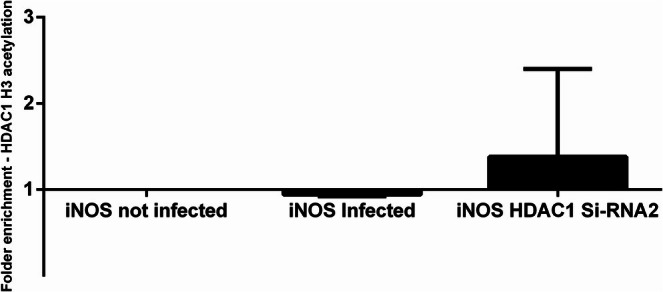
Analysis of histone acetylation in the proximal promoter region *of* iNOS in response to *Leishmania infantum* infection. DH82 cells (2 × 10^5^ cells/mL) were infected with *L. infantum* for 6 h. The pattern of histone acetylation at the iNOS promoter was analysed by ChIP. Antibodies specific for H3 acetylation. Changes in H3 acetylation and HDAC1 occupancy are shown for uninfected cells (UI). Basal levels of H3 acetylation and occupancy in uninfected cells were used for data normalization and to assess relative abundance. Numbers > 1 indicate upregulation, and < 1 indicate downregulation of host genes in infected cells compared to uninfected cells. Results are the mean and standard error of the mean (*n* = 2).

The ChiP assay evaluated histone acetylation in the iNOS defence gene. Chromatin immunoprecipitation was performed with chromatin from DH82 cells infected with *L. infantum* or transfected with siRNA2, after which qRT‐PCR was performed to analyse iNOS expression. We observed a decrease in Ac‐H3 acetylation at the iNOS promoter in infected cells compared to uninfected cells. The level of iNOS transcripts in infected cells was lower compared to uninfected cells. Inhibition of the enzyme by Si‐RNA2 led to increased iNOS expression in the gene's promoter region (Figure 4).

### 3.5 Pharmacological Inhibition of HDAC Decrease Expression of IL‐6, TNF‐α and IL‐10

**FIGURE 5:**
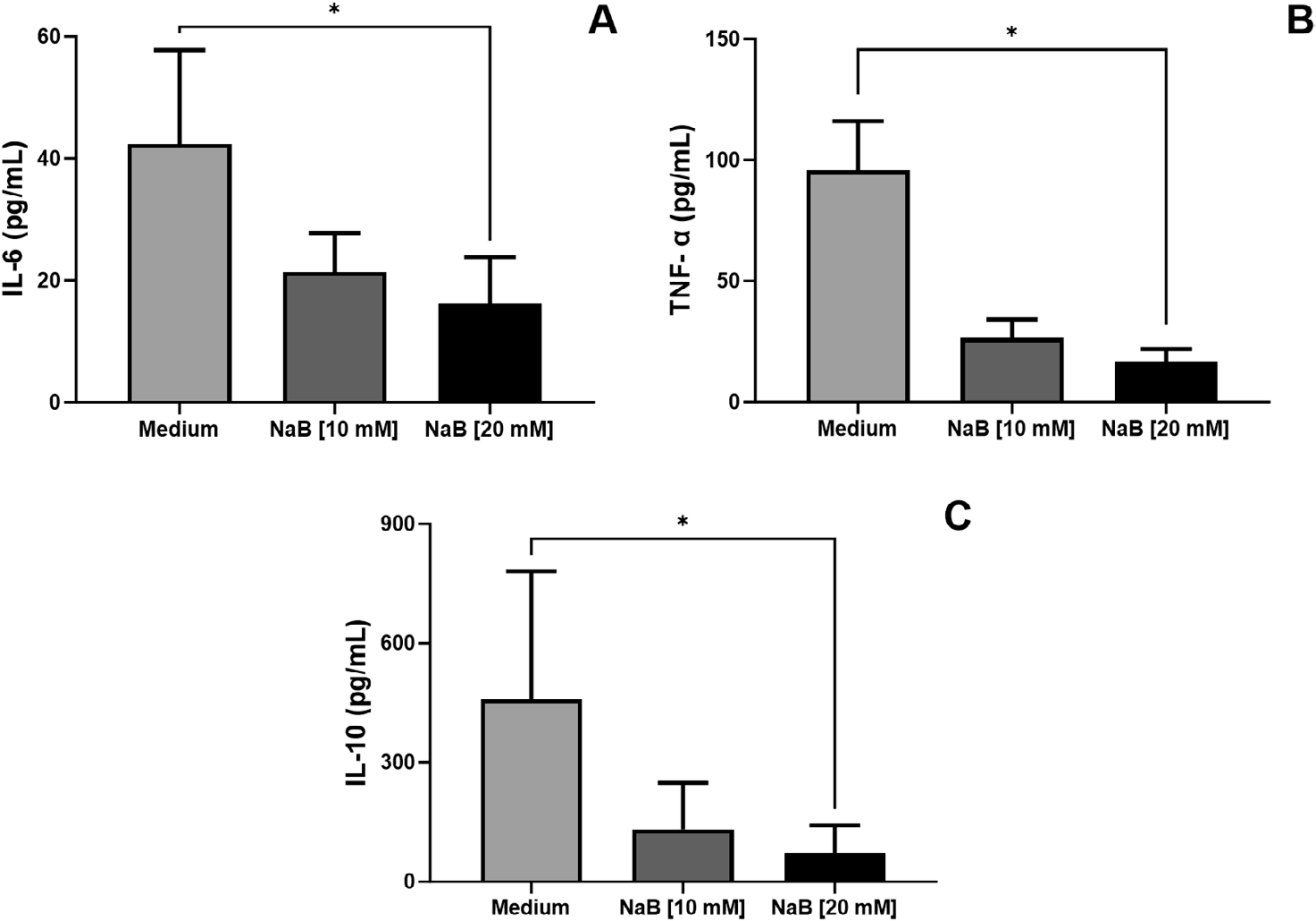
Inhibition of HDAC1 in cells infected with *Leishmania infantum* and treated with sodium butyrate on IL‐6, TNF‐α and IL‐10 expression. DH82 cells were treated with the pharmacological HDAC1 inhibitor sodium butyrate NaB (10 mM) and (20 mM). After 6 h of infection, the culture supernatant cells were harvested, and (A) IL‐6, (B) TNF‐α and (C) IL‐10 were measured by capture ELISA. The statistical test used was the Friedman test, followed by Dunn's multiple‐comparison test (*p* < 0.05).

As histone acetylation is crucial for regulating NF‐kB‐mediated inflammation during *L. amazonensis* infection [23], we assess whether HDAC1 and HDAC2 are involved in the expression of pro‐ and anti‐inflammatory cytokines after *L. infantum* infection in NaB‐treated cells. We observed decreased IL‐6, TNF‐α and IL‐10 levels in culture supernatant from NaB (20 mM) treated cells, IL‐6 (42.3 ± 27.6 (Medium), 21.3 ± 9.3 (NaB [20 mM]), Friedman test, followed by Dunn's multiple‐comparisons test, *p = 0.0342), TNF‐α (95.5 ± 29.9 (Medium), 16.8 ± 7.0 (NaB [20 mM]), Friedman test, followed by Dunn's multiple‐comparisons test, *p = 0.0140), IL‐10 (458.6 ± 514.7 (Medium), 73.1 ± 108.9 (NaB [20 mM]), Friedman test, followed by Dunn's multiple‐comparisons test, *p = 0.0133) (Figure 5A–C).

## 4 Discussion

Chromatin is recognised as an essential target for many pathogens. In parasitic infection, there is epigenetic modulation in host cells [31, 32]. Histone deacetylases mediate chromatin remodelling and the silencing of host genes essential for defence against the parasite [33]. We therefore set out to assess whether parasite infection modulates HDAC1 in canine macrophages and regulates their microbicidal activities and cytokine production. We observed increased HDAC1 expression in DH82 cells infected with *L. infantum*, and HDAC1 inhibition decreased parasite load, increased iNOS in macrophages and modulated cytokine production.

Our results showed increased HDAC1 expression in DH82 cells infected with *L. infantum* compared to uninfected cells. Similar studies showed increased HDAC1 expression in THP‐1 cells derived from human macrophages infected with *L. donovani* [17] and with *L. amazonensis*. HDAC1 modulates infection in different *Leishmania* species. In addition, these mechanisms have already been observed in THP‐1 cells infected with *Mycobacterium tuberculosis* [34], suggesting that this response is not specific to *Leishmania* infection and that other intracellular pathogens also modulate epigenetic processes to favour infection.

Histone deacetylase inhibitors are being investigated as drugs for various diseases, including parasitic diseases [18]. Sodium butyrate inhibits HDAC1 and HDAC2 [27]. We observed that parasite load decreased in DH82 cells after *L. infantum* infection when treated with the pharmacological inhibitor sodium butyrate; a similar result was observed in THP‐1 cells infected with *L. donovani* [17]. Also in vivo, pharmacological inhibition of HDAC in BALB/c mice infected with *L. braziliensis* effectively reduced lesion size and parasite burden [35]. Together, these findings support the notion that HDAC inhibition consistently reduces *Leishmania* burden across different host models and species. This reinforces the role of HDAC activity in promoting parasite survival and highlights HDAC inhibitors as promising candidates for adjunct therapeutic strategies against leishmaniasis.

Our results showed decreased parasite load in cells silenced with HDAC1‐siRNA1 and HDAC1‐siRNA2. A similar study showed a reduction in parasite load in THP‐1 cells infected with *L. amazonensis* upon HDAC1 silencing [26], suggesting that HDAC1 inhibition may regulate immune response genes involved in parasite control. Low levels of HDAC1 are associated with lower amastigote concentrations within macrophages, possibly due to increased iNOS production and reduced parasite survival in host cells.

In leishmaniasis, NO production by iNOS in murine macrophages is crucial for controlling *Leishmania* at all stages of infection [7]. In canine leishmaniasis, decreased NO expression in splenic cells of dogs with disease symptoms [10] and increased iNOS expression in canine macrophages are associated with decreased parasite load [30]. We observed that silencing with HDAC1‐siRNA1, HDAC1‐siRNA2 or NaB increased iNOS levels and expression in DH82 cells infected with *L. infantum*; similar findings were observed in THP‐1 cells infected with *L. amazonensis* and silenced with ShHDAC1 [26]. Evidence also suggests that HDAC1 inhibition with NaB increases the expression of genes encoding defensins and myeloperoxidase [17], proteins associated with leishmanicidal activities [17]. Inhibition likely increased histone acetylation, thereby stimulating iNOS production [36], a defence mechanism against visceral leishmaniasis [17]. Therefore, we suggest that iNOS expression is also regulated by HDAC1 in the canine model.

Furthermore, in human THP‐1 cells infected with *Mycobacterium tuberculosis* when HDAC1 was inhibited with butyrate, there was an increase in the concentration of IL‐12 [34], in THP‐1 cells infected with *L. donovani* , HDAC1 inhibition increases the expression of CIITA (IFN‐ƴ responsive gene) [37]; similar results were observed in murine macrophages infected with *T. gondii* [38], suggesting that silencing and inhibition of HDAC1 may modulate initial defence mechanisms against intracellular pathogens. Future studies will be essential to clarify other HDAC1‐regulated factors that may also act in the canine model and regulate iNOS expression. Furthermore, it is important to note that the results are from an in vitro model; in vivo studies should be conducted to confirm them.

In our studies, we observed increased HDAC1 expression in cells infected with *L. infantum*, and that silencing or pharmacological inhibition of HDAC1 led to increased iNOS expression and levels. These results led us to hypothesise that the hypoacetylation of H3, a consequence of decreased HDAC1 during infection, may alter the transcription of the defence gene iNOS. Previously, it was shown that *L. donovani* infection in human cells decreases global H3 acetylation in genes responsive to interferon γ (IFN‐γ) [37]. It is known that IFN‐γ is an essential cytokine that stimulates macrophages to induce iNOS expression and NO synthesis, leading to the death of the intracellular parasite [7].

We observed negative regulation of histone H3 acetylation at the iNOS gene promoter in DH82 cells after 6 h of infection. As observed, *L. amazonensis* substantially reduces H3 acetylation and methylation at sites of inflammatory gene activation, making chromatin less accessible and promoting parasite survival [23].

In addition, HDAC1 and p50/p50 NF‐κB form the same repressor complex at the iNOS promoter during *L. amazonensis* infection [26]; furthermore, activation of the NF‐κB pathway has been shown to increase iNOS expression in murine cells infected with *L. donovani* [39], suggesting that further studies can be carried out with this promoter in DH82 cells to clarify the mechanisms involved.

Cellular reprogramming to stimulate iNOS production by macrophages depends on the stimulation of inflammatory cytokines [40]. The role of HDAC1 in modulating these cytokines during *L. infantum* infection is still limited. In this study, IL‐6, TNF‐α and IL‐10 decreased after pharmacological inhibition of HDAC1 and HDAC2; similarly, NaB potently suppressed the expression and secretion of pro‐inflammatory cytokines from classically activated M1 BMDM and RAW264.7 cells [41]. HDAC1 may regulate inflammatory cytokines such as IL‐12 and TNF‐α in THP‐1 cells infected with *L. donovani* [42]. These results indicate that HDAC1‐mediated deacetylation of histone H3 during infection may be an important epigenetic mechanism for the parasite's survival in the host cell. Future studies should investigate how HDAC1 regulatory mechanisms influence the balance between pro‐inflammatory and anti‐inflammatory cytokines in vivo.

However, the possibility that other mechanisms may be acting alongside HDAC1 to facilitate parasite evasion in the host cell cannot be ruled out. In human macrophages infected with *Leishmania donovani*, DNA methylation at CpG sites can inactivate genes responsible for the immune response, contributing to the progression of the infection [43]. Furthermore, non‐coding microRNAs appear to inhibit gene regions that regulate the inflammatory response and the production of iNOS and NO in canine visceral leishmaniasis [44, 45], which enhances the survival and replication of the parasite. Future studies will be needed to assess how these mechanisms, together, may be modulating the host's response.

It is worth noting that this is the first study to evaluate H3 and HDAC1 acetylation in canine macrophages infected with *L. infantum*. In conclusion, the epigenetic regulation of iNOS expression and cytokine production by HDAC1, associated with a decrease in parasite load, provides an essential mechanism for modulating the innate immune response and establishing infection in canine macrophages. Although the studies were conducted in vitro, further research will be essential to validate the findings in vivo.

## Author Contributions

Gabriela Lovizutto Venturin performed experiments, data acquisition, analysis, interpretation and drafted the manuscript. Lucas Takeshi Siqueira Ito, Gisele Mitsue Umino, Gabriela Torres Rebech and Flávia Lombardi Lopes assisted in the experiments and analysed data. Valéria Marçal Felix de Lima was responsible for project guidance, analysis, interpretation and final drafting of the manuscript. All authors have read and approved the final manuscript.

## Conflicts of Interest

The authors declare no conflicts of interest.

## Supporting information


**Figure S1:** Microscopic slides prepared after in vitro infection with *Leishmania*, where *Leishmania infantum* was observed inside DH82 cells. Infection was examined at oil‐immersion magnification on an Olympus BX61 microscope equipped with a DP71 camera.

**Figure S2:** A representative overlay histogram illustrates HDAC1 expression in DH82 cells, not infected and infected with *L. infantum*.

**Figure S3:** Expression of HDAC1 in DH82 cells infected with *L. infantum* and transfected with (A) Sc‐siRNA and HDAC1‐si‐RNA1, (B) Sc‐siRNA and HDAC1‐si‐RNA2 and (C) DH82 cells inhibited with NaB (10 mM). The statistical test used was Wilcoxon (*p* < 0.05). The graphs are represented with the mean and standard error of the mean.

**Figure S4:** Representative slides of infection index showing *Leishmania infantum* inside DH82 transfected with (A) Sc‐siRNA, si‐RNA1 and si‐RNA2 and (B) DH82 cells inhibited with NaB (10 mM) and (20 mM).

**Figure S5:** A representative overlay histogram shows an example of iNOS production in DH82 cells infected with *L. infantum*, transfected with (A) HDAC1‐si‐RNA1 (B) HDAC1‐si‐RNA2 and (C) inhibited with NaB (10 mM).

## Data Availability

The data that support the findings of this study are available from the corresponding author upon reasonable request.
