# Supplementary figures and images for: The Regulatory Effect of Histone Deacetylase (HDAC) 1 and 2 on iNOS, IL‐6, TNF‐α and IL‐10 Expression in Canine Macrophages Infected With Leishmania infantum
Source: Parasite Immunol. 2026 Apr 6;48(4):e70078. doi: 10.1111/pim.70078 (PMC13051526; doi:10.1111/pim.70078)

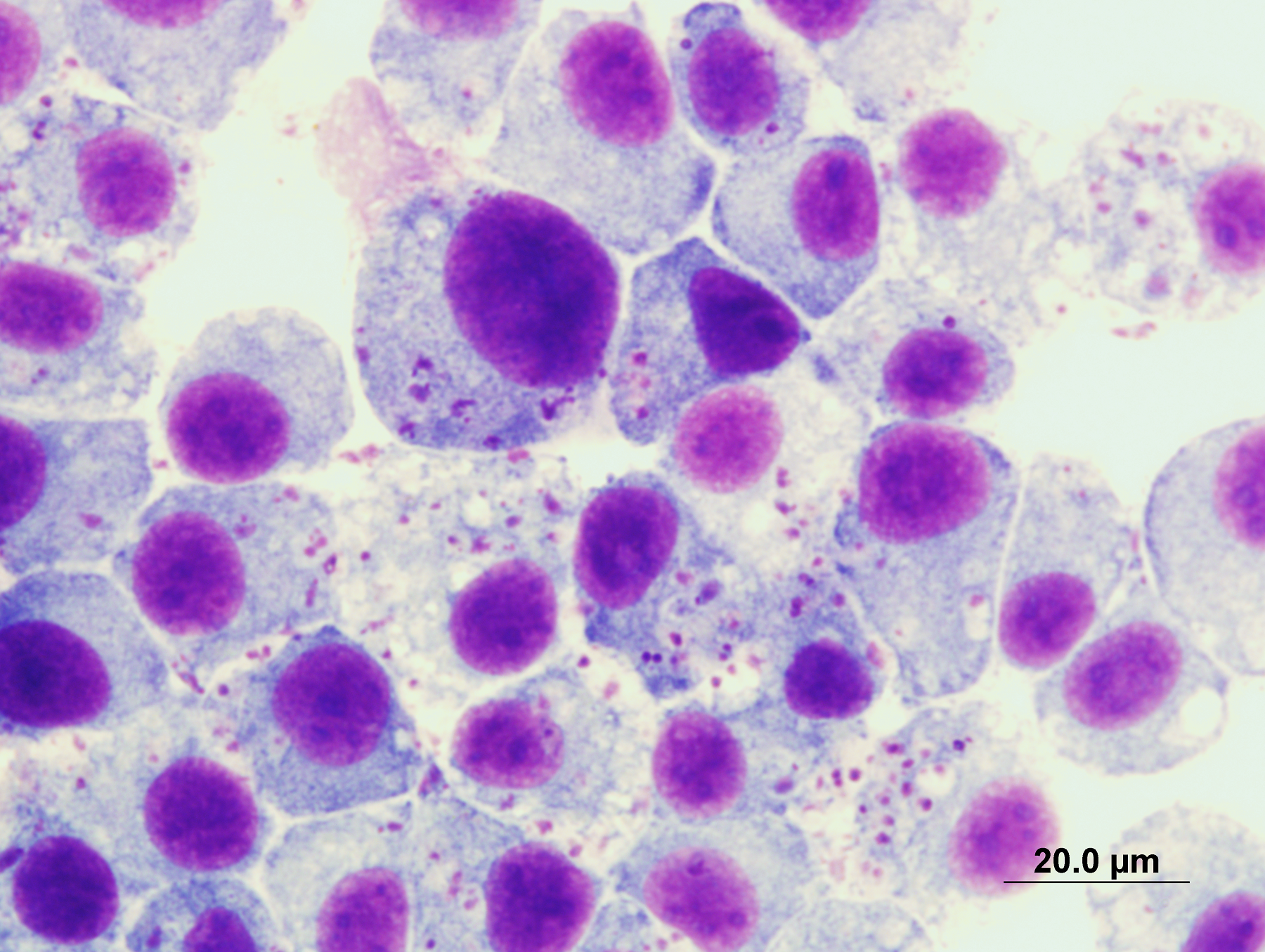

Supplement: Supplementary file 1 — Figure S1: Microscopic slides prepared after in vitro infection with Leishmania, where Leishmania infantum was observed inside DH82 cells. Infection was examined at oil‐immersion magnification on an Olympus BX61 microscope equipped with a DP71 camera. [file PIM-48-e70078-s003.tif]

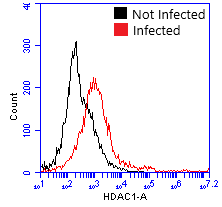

Supplement: Supplementary file 2 — Figure S2: A representative overlay histogram illustrates HDAC1 expression in DH82 cells, not infected and infected with L. infantum. [file PIM-48-e70078-s005.tif]

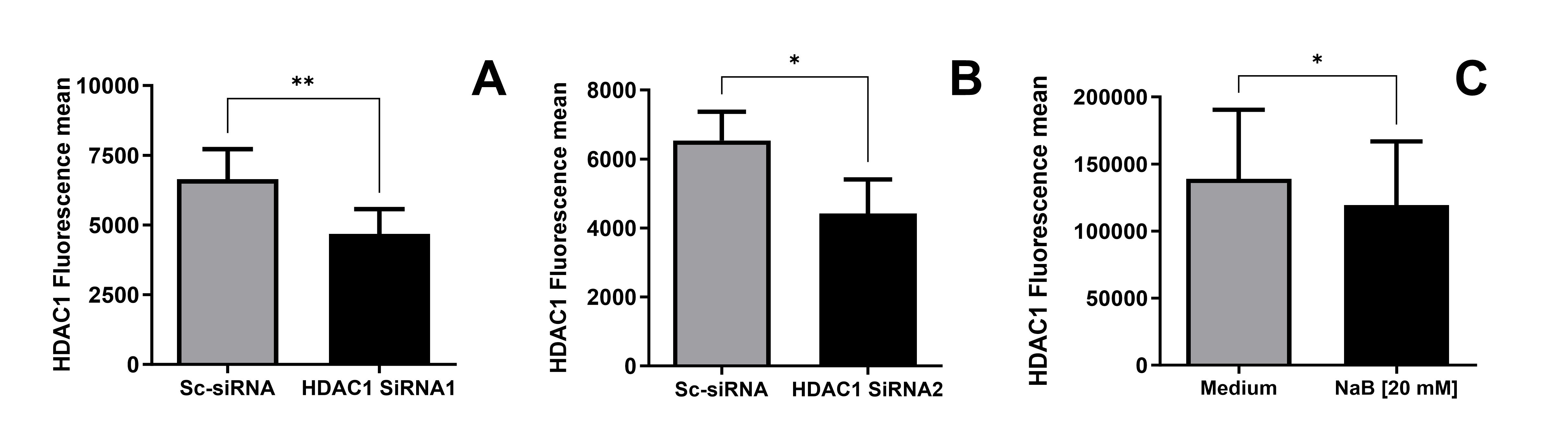

Supplement: Supplementary file 3 — Figure S3: Expression of HDAC1 in DH82 cells infected with L. infantum and transfected with (A) Sc‐siRNA and HDAC1‐si‐RNA1, (B) Sc‐siRNA and HDAC1‐si‐RNA2 and (C) DH82 cells inhibited with NaB (10 mM). The statistical test used was Wilcoxon (p < 0.05). The graphs are represented with the mean and standard error of the mean. [file PIM-48-e70078-s001.tif]

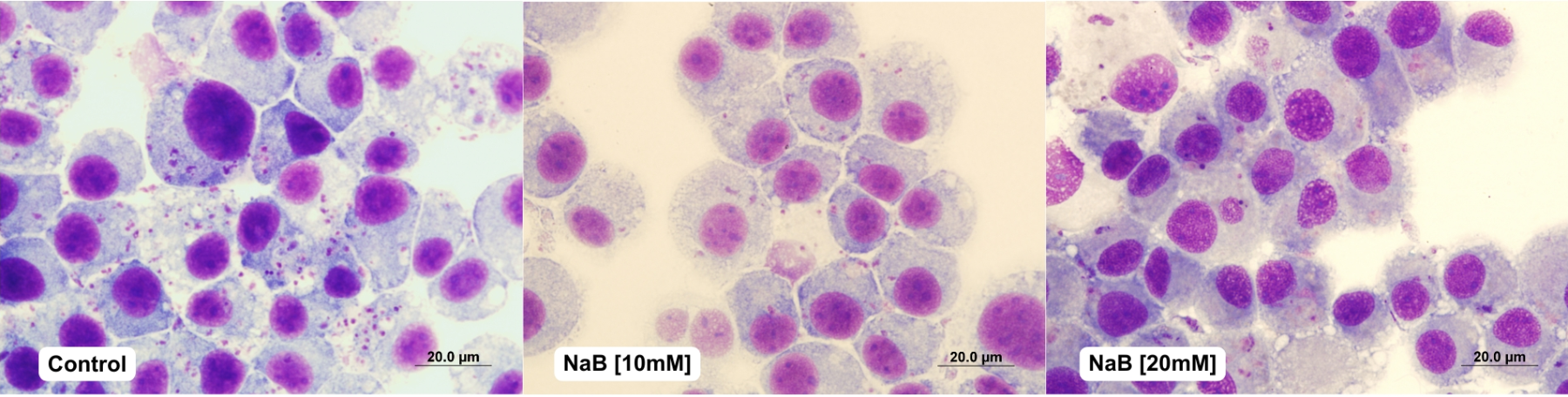

Supplement: Supplementary file 4 — Figure S4: Representative slides of infection index showing Leishmania infantum inside DH82 transfected with (A) Sc‐siRNA, si‐RNA1 and si‐RNA2 and (B) DH82 cells inhibited with NaB (10 mM) and (20 mM). [file PIM-48-e70078-s004.zip › pim70078-sup-0003-Supinfo3@Suporting information 4B.tiff]

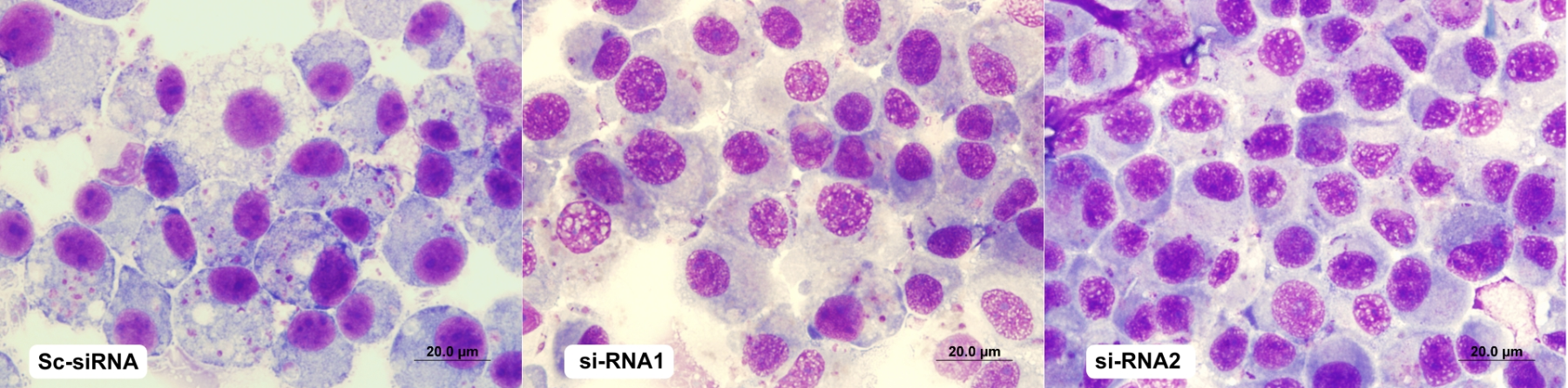

Supplement: Supplementary file 4 — Figure S4: Representative slides of infection index showing Leishmania infantum inside DH82 transfected with (A) Sc‐siRNA, si‐RNA1 and si‐RNA2 and (B) DH82 cells inhibited with NaB (10 mM) and (20 mM). [file PIM-48-e70078-s004.zip › pim70078-sup-0002-Supinfo2@Suporting information 4A.tiff]

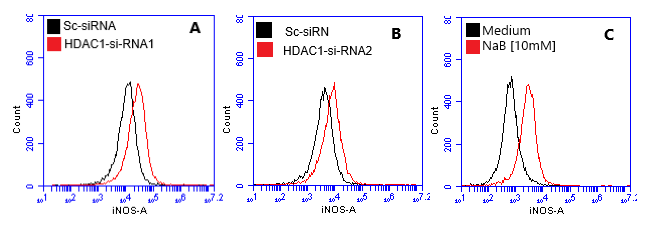

Supplement: Supplementary file 5 — Figure S5: A representative overlay histogram shows an example of iNOS production in DH82 cells infected with L. infantum, transfected with (A) HDAC1‐si‐RNA1 (B) HDAC1‐si‐RNA2 and (C) inhibited with NaB (10 mM). [file PIM-48-e70078-s002.tif]
